# Brain Maturation as a Fundamental Factor in Immune-Neurovascular Interactions in Stroke

**DOI:** 10.1007/s12975-022-01111-7

**Published:** 2023-01-27

**Authors:** Elena Di Martino, Aditya Rayasam, Zinaida S. Vexler

**Affiliations:** Department of Neurology, University California San Francisco, 675 Nelson Rising Lane, San Francisco, CA 94158-0663 USA

**Keywords:** Neonatal stroke, Childhood arterial stroke, Inflammation, Blood–brain barrier, Choroid plexus

## Abstract

Injuries in the developing brain cause significant long-term neurological deficits. Emerging clinical and preclinical data have demonstrated that the pathophysiology of neonatal and childhood stroke share similar mechanisms that regulate brain damage, but also have distinct molecular signatures and cellular pathways. The focus of this review is on two different diseases—neonatal and childhood stroke—with emphasis on similarities and distinctions identified thus far in rodent models of these diseases. This includes the susceptibility of distinct cell types to brain injury with particular emphasis on the role of resident and peripheral immune populations in modulating stroke outcome. Furthermore, we discuss some of the most recent and relevant findings in relation to the immune-neurovascular crosstalk and how the influence of inflammatory mediators is dependent on specific brain maturation stages. Finally, we comment on the current state of treatments geared toward inducing neuroprotection and promoting brain repair after injury and highlight that future prophylactic and therapeutic strategies for stroke should be age-specific and consider gender differences in order to achieve optimal translational success.

## Introduction

The dynamic nature of brain development plays a key role in injurious cascades following an ischemic event and affects the magnitude and evolution of brain damage. The postnatal brain maturation stage and the unique pace of individual cell type development in the Central Nervous System (CNS) at the time of stroke leads to distinct excitotoxic and inflammatory signatures. Thus, it is of fundamental importance to consider targeting pathophysiological pathways in maturation-specific ways to preserve brain function and promote repair of the developing brain after stroke. In this review, we summarize both predisposing and modulatory factors that determine the extent of maturation-dependent ischemic brain injury and describe the inflammatory and molecular patterns related to immune-neurovascular interactions.

## Brain Development as a Potential Modifying Factor in Brain Injury

Development of the human brain is a precisely orchestrated process that requires careful synchronization between maturation of CNS barriers and cells within the brain parenchyma. In humans, brain development commences when neuroepithelial cells of the ectoderm give rise to the neural plate and closure of the neuronal tube that initiates the formation of the CNS in gestational week 3 (GW3) [1], followed by separation of the CNS from the periphery by the brain vasculature by GW5 [2] and subsequent formation of the choroid plexus (CP) around GW7 [3] and most of the meningeal structures at GW12 [4]. Tight junction (TJ) proteins, such as occludin and claudin-5, which are the structural foundation of the CNS barriers, are detected in the fetal brain at GW16 [5]. Pyramidal neural cells derive from the proliferating progenitor cells, such as radial glial cells, as early as GW5 [2]. Neurogenesis, which continues through early postnatal life [6], peaks between GW9-13 [7], with newly formed neurons migrating radially from the subventricular zone (SVZ) to the cortex [2]. Synaptogenesis begins as early as GW8 [8] with the neuronal circuits shaped by synaptic pruning by microglia and astrocytes [9]. Microglia are found in the brain before vascular sprouting, as early as GW5 [10, 11], whereas astrocytes appear later, by GW15 [12], as are oligodendrocyte (OL) progenitor cells, by GW17 [13, 14]. Both microglia [15] and pre-OLs [13, 14] continue to differentiate overtime. Myelination starts during mid-gestation and continues until late adolescence [16].

**Fig. 1:**
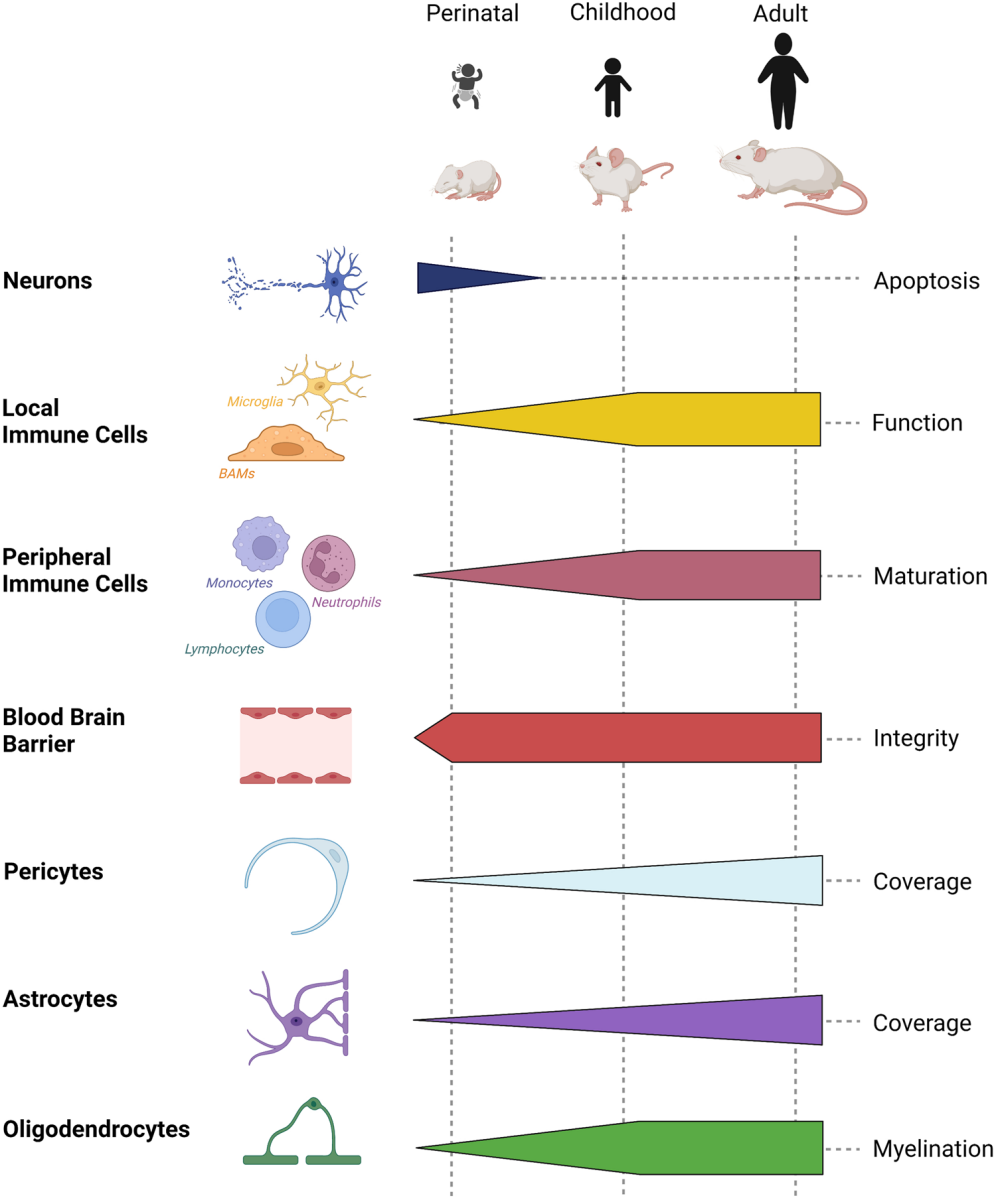
Schematic representation of the development of individual cell types and of the maturing neurovascular unit during embryonic and postnatal brain development in rodents. Vertical punctate lines indicate approximate time for inducing tMCAO to mimic PAIS, CAIS, and AIS. As is evident from this cartoon presentation, physiological neuronal apoptosis (i.e., programmed cell death) is high in the newborn brain and rapidly declines during perinatal period. Microglial function undergoes changes with development. Peripheral cells are immature in neonatal mice and reach maturation during juvenile period. The BBB is established by birth but continues to change in neonatal and juvenile brain. Astrocyte and pericyte coverage continues to increase in neonatal and juvenile brain. Myelination begins during postnatal period and is complete in juvenile rodents. Image created with BioRender.com

**Fig. 2:**
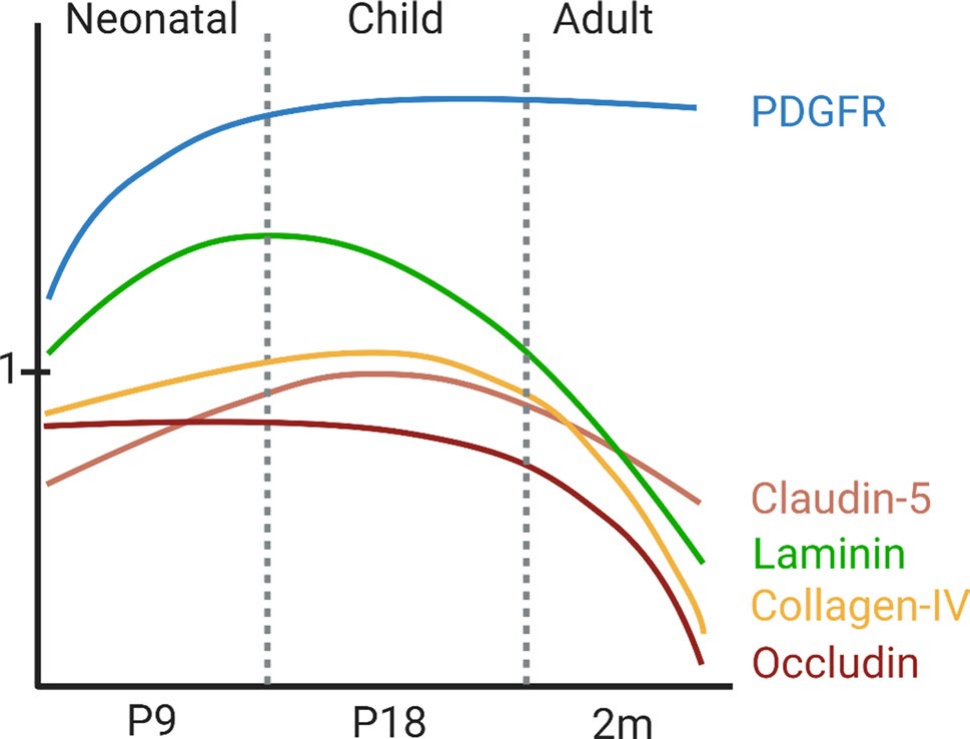
Developmental changes in expression of individual TJ proteins during postnatal days P9–P60. On Y axes, 1 represents expression of individual proteins in P7 rat brain based on Western Blot data published in [108]

In rodents, maturation of the blood-brain barrier (BBB) was shown to intimately relate to recruitment of endothelial cells and pericytes into the brain [17–19], a process that commences around embryonic day (E) 11. Permeability of BBB endothelial cells is tightly restricted by E15 in mice [20], prior to astrogliogenesis, which begins closer to birth [21]. The TJ protein zonula occluden protein 1 (ZO-1) starts to be expressed in E15 cerebral vessels in the mouse, and by E19, the TJ appears completely differentiated [22], demonstrating that astrocytes do not contribute to the early induction of “tightness” of the BBB. Figure 1 demonstrates the unsynchronized changes in individual components of the BBB during fetal and postnatal physiological brain development. Figure 2 details changes in expression of TJ proteins during postnatal day (P)9–P60. VEGFR2 deficiency prohibits vessel formation, leading to embryonic lethality [23]. In contrast to astrocytes, microglial cells have direct effects on vasculogenesis and vascular sprouting in the embryonic brain [24, 25] by guiding endothelial sprouts, largely via VEGF-dependent mechanisms. Microglial deficiency adversely affects vasculature development [26], and monocytes cannot substitute for the lack of microglia in vasculogenesis in the developing brain [24]. Disrupted integrin and chemokine signaling also adversely affect embryonic angiogenesis and BBB formation. Microglia also regulate embryonic and postnatal physiological brain development via secretion of trophic factors, immuno-surveillance, oligodendrogenesis, and neurogenesis and ultimately establish brain connectivity network during postnatal brain development [27–29]. Therefore, the timing of these developmental cues in relation to maturation of individual cell types of the neurovascular unit is critical in influencing the pathophysiology of cerebral ischemia depending on the stage of brain development.

## Epidemiology of Neonatal (Perinatal) and Childhood Stroke

Stroke in the neonatal/perinatal period (i.e., between gestational week 20 to the first 28 days of life) could manifest acutely or in a delayed form, can be arterial or venous, and present as ischemic or hemorrhagic [30]. The incidence of neonatal stroke is 1 per 2300–5000 births, and it commonly presents with seizures, encephalopathy, neurological deficits, and is typically diagnosed by MRI [31–35]. Estimates from 2015 indicate that the overall under 5-year-old mortality rate following ischemic events in infants and children is 43 per 1000 live births, of which 45% of the deaths occur during the neonatal period [36]. Gestational diabetes, preeclampsia, and chorioamnionitis are considered risk factors that may affect stroke occurrence [37], while systemic infection, pre-term brain injury, asphyxia, and stroke during the perinatal period all include inflammatory components [38].

Compared to perinatal arterial ischemic stroke (PAIS), childhood AIS (CAIS) has a distinct pathophysiology. The incidence of CAIS is low, 1–2 children per 100,000 [39], but its recurrence is high, making it one of the top 10 causes of pediatric mortality [40, 41]. Recent cohort studies have reported that childhood stroke is associated with long-term mortality, even 20 years after stroke [42]. Furthermore, age at the time of the insult negatively affects long-term cognitive and motor outcomes and increases the risk of attention deficit/hyperactivity disorder (ADHD) in older children [43–45]. One unique discovered aspect of CAIS is that it frequently occurs shortly after viral infection [46], such as Varicella Zoster Virus (VZV) [47], and virus-induced immune-related cerebral arteriopathies [48].

## Models of Neonatal and Childhood Stroke

Age that approximates the human brain at term is species-dependent (reviewed in [49–51]). As an example, sheep are precocial, and thus, to relate to human brain development, hypoxia studies are performed during pregnancy, and transient occlusion of the umbilical cord prior to birth is used in non-human primates to model severe asphyxia [52]. Other commonly used non-rodent large mammal species to induce ischemia-related brain injury is performed in rabbits and pigs. Species that have a white/gray matter ratio similar to the human brain and pigs in particular are useful for monitoring post-stroke cerebral blood flow (CBF) changes, but these models mimic hypoxia–ischemia (HI).

In rats and mice, most brain development occurs after birth like humans, yet the developmental growth of individual brain regions is distinct in rodents and humans, thus making it difficult to adhere to a single postnatal day as a comprehensive representation of brain development in humans. Cross-comparisons of gross neuroanatomy, the timing of neurogenesis, synaptogenesis, gliogenesis, maturation, and myelination as well as age-dependent molecular and biochemical changes in rodents and humans have demonstrated that the rodent brain at P1–P5 corresponds to 23–32 weeks of gestation in humans and is thus suitable for studies of preterm injury, whereas the rodent brain at P7–P10 corresponds to 36–40 weeks of gestation in humans, thus suitable for studying brain injury close to or at term (reviewed in [53]). Brain myelination in rodents is completed between P17 and P25, and brain maturation is thought to correspond to that of a toddler.

For quite some time, all data regarding ischemia-related pathology in the neonatal rodent brain was based on the Rice-Vannucci HI model in P7 rats, which involves unilateral/bilateral ligation of the common carotid artery followed by systemic hypoxia of variable length and oxygen deprivation [54–56], and in P9 mice (reviewed in [51, 57, 58]). Due to the presence of systemic hypoxia, however, the HI model is more representative of hypoxic-ischemic encephalopathy (HIE) and not focal stroke (reviewed in [59, 60]).

A different group of models was developed to examine the pathophysiology of neonatal stroke by using middle cerebral artery occlusion (MCAO), the most common type of ischemic stroke in at term infants. Transient MCAO (tMCAO) was achieved in P17 rats [61] or P7 rats [62] or via permanent left middle cerebral artery occlusion in association with occlusion of the left common carotid artery [63]. Additional tMCAO models of different durations were developed in P10 rats [64] and P9–P10 mice [65]. Varying duration of MCAO between 1.5 and 3 h enabled injury of different severities [65–67]. MRI during MCAO and after retraction of suture filament demarcated ischemic injury in the vascular territory of the MCA with a definable ischemic core and penumbra [68, 69] and demonstrated injury evolution over time [70–74].

CAIS has different etiologies, risk factors, and presentation compared to either neonates or adults [39, 46, 75]. To understand the pathophysiology of CAIS, models of MCAO or endothelin-1 injection were developed in juvenile (P17–P25) rodents [61, 76–78]. As we discuss below, comparative studies between newborn, juvenile, and adult rodents subjected to various excitotoxic and inflammatory conditions further extended the concept of a critical role of maturational stage in influencing stroke pathology. These models include intracortical IL-1β injection [79, 80], brain trauma [81–83], and ischemic arterial stroke [77, 78, 84]. In addition, to examine the role of infection in ultimately predisposing childhood brain to stroke, we developed a model of viral-like infection (viral mimetic Poly-IC) in P18 mice and demonstrated infection-induced arteriopathy [85]. Nonetheless, experimental studies of CAIS remain sparse and molecular mechanisms of susceptibility to CAIS and its recurrence are insufficiently defined.

## Pathophysiology and Early Mechanisms of Injury

There are both common and distinct mechanisms between the pathophysiology of PAIS and CAIS related to particular circumstances that occur around the time of birth (neonates) such as evolving myelination and modulation of mitochondrial function (children).

### Cerebral Blood Flow, Energy Metabolism, and Cell Death

Cerebral ischemia rapidly disrupts brain homeostasis in both the adult and immature brain, leading to generalized shutdown of ATP-dependent processes; increased anaerobic glycolysis; Na + /K + pump malfunction; Na^+^, Cl^−^, and H2O influx; cytotoxic edema; neuronal membrane destabilization; glutamate release; stimulation of NMDA/AMPA receptors to massively release intracellular Ca2^+^ ions; and ensuing mitochondrial damage. These processes are very nuanced depending on the age due to multiple factors. Early collateral recruitment after stroke differs in infants and adults [86]. In rodents, the brain vasculature undergoes extensive endothelial proliferation and branching in the first postnatal month. Endothelial cell proliferation peaks around P10 [87], and vascular density increases from P8 to peak at P21 [88]. NMDA receptors undergo changes during brain development [89]. These factors, together with maturational switch from predominant glycolysis to oxidative phosphorylation during the second postnatal week in rodents, play an important role in energy deprivation, phenomena extensively studied in neonatal HI model (reviewed in [90–92]). Altogether, oxidative damage and excitotoxicity instigate microvascular injury, triggering robust post-ischemic inflammation driven by resident and peripheral immune cells, signaling that can trigger both necrotic and apoptotic cell death [90, 93–96]. Apoptotic pathways are more readily activated following tMCAO in neonates compared to adults due to expressed proteins involved in developmental programmed cell death [69, 97, 98], processes more prominent in the penumbra than in the ischemic core of neonatal rodents. Importantly, sex-related differences in apoptotic pathways were identified after neonatal cerebral ischemia [99, 100]. Other types of cell death have been described in HI models, autophagy and ferroptosis, but those have not been studied in arterial ischemic stroke models. Oxidative stress was demonstrated to influence collateral flow of reperfused vessels following ischemic events in cerebral vessels of post-ischemic neonatal and juvenile brain [101], but oxidative stress mechanisms are yet to be defined in CAIS models.

### The Brain Barrier Interfaces

The brain is protected by multiple barrier systems that include the BBB, the blood-cerebrospinal fluid barrier (BCSFB) in the CP, and the leptomeningeal CNS barrier [102, 103]. During pathological conditions, altered BBB functionality and peripheral immune cells undermine the isolated nature of the CNS [104, 105]. The breakdown of the BBB is well characterized after adult stroke [106, 107], whereas emerging evidence has suggested that the early postnatal BBB is not as permeable as the adult BBB after an ischemic insult [104, 108]. Studies in a model of tMCAO showed that albumin leakage into injured regions was markedly increased during 2–24h reperfusion in adult rats compared to P7 rats, whereas expression of collagen-IV, laminin, claudin-5, occludin, and ZO-1 was relatively low [108]. Infiltration of peripheral immune cells into the acutely injured neonatal brain was also limited in neonates. The CP has lately been identified as the gate for lymphocyte trafficking and entry to the parenchyma under both physiological and pathological circumstances [109]. In fact, the CP senses change in the CSF to alter circulating immune cells [110] as well as recruit inflammatory cells of myeloid lineage to the damaged area in models of CNS injuries [111, 112]. In a neonatal mouse stroke model, robust accumulation of myeloid cells, including neutrophils as well as inflammatory and beneficial monocytes, has been identified in the CP ipsilateral to injury [105], likely contributing to inflammatory injury component in the parenchyma. The meninges have been shown to support brain development and serve as an important site of immune cell expansion and reactivity during early phases of immune response after preterm brain injury [113], but the role of the meninges in stroke in the immature brain is relatively unexplored.

BBB permeability differs in a rodent CAIS model compared to both PAIS and AIS models [78, 108]. BBB disruption is also much higher in juvenile that in newborn or adult rats following intracerebral IL-1β administration [79], with distinctions likely due to myelination and leukocyte maturation during postnatal development (Fig. 1). Furthermore, in the mouse brain, cortical vessel branching reaches a plateau between P15 and P25, which could affect the extent in collateral flow and stroke severity [86]. Systemic immune activation in juveniles can also promote procoagulant effects and local inflammation to induce fragility of cerebral arteries and yield the juvenile brain susceptible to subsequent stroke [114].

### The Neurovascular Unit

Endothelial cells (ECs) are very sensitive to oxidative and inflammatory processes. TJ damage due to post-ischemic ROS accumulation can lead to EC dysfunction and death [115, 116], gradual loss of EC proliferative capacity, dysfunctional endothelial progenitor cell (EPC) migration, and impaired secretion of growth factors [117]. VEGF receptor inhibition after neonatal stroke was shown to reduce EC proliferation [118, 119]. Furthermore, transcriptional analysis of ECs isolated from adult and neonatal rat brains 24 h after tMCAO revealed a strikingly different gene regulation pattern among ages, where neonates had a better preserved expression of occludin, claudin-5, and ZO-1 [108], thus highlighting age-specific signaling between adults and neonates in neurovascular and immune cells after brain injury. ECs were recently reported to directly cross-talk with perivascular macrophages (PVMs) and shift their phenotype to anti-inflammatory in support of the BBB in the adult brain [120].

**Fig. 3:**
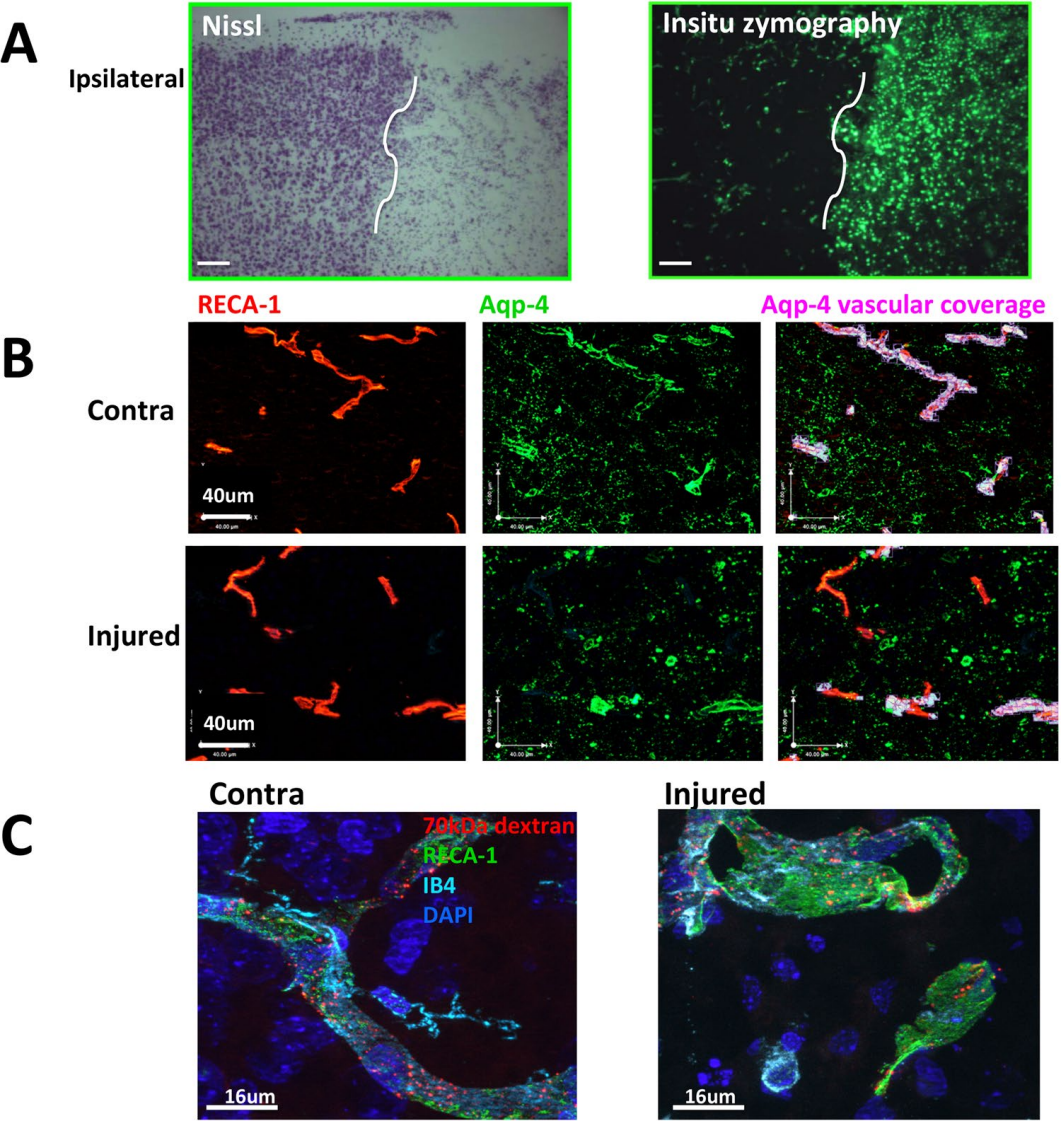
Examples of effects of 3h MCAO followed by 24h reperfusion in P7 rats on the ECM degradation (**A**), vascular Aqp-4 coverage (**B**), and BBB integrity (**C**). **A** In situ zymography in coronal section (right) shows ECM degradation in the penumbral and ischemic core regions, regions defined by Nissl staining (left). **B** Aqp-4 coverage of vessels in contralateral region (top) and ischemic-reperfused region (bottom). Note that Aqp-4 expression is reduced and detracted from the vessels in ischemic-reperfused region. **C** 70-kDa Dextran administered 23 h after reperfusion is observed within vessels in both contralateral (image on the left) and the injured cortex (image on the right)

The extracellular matrix (ECM)/basement membranes provide structural support to cells but also serve as reservoir of growth factors that direct and fine-tune cellular functions. Type IV collagens are a major component of all basement membranes which, together with laminins, play a major regulatory role in determining the molecular stoichiometry. Mutations in the α1(IV) chain (or COL4A1) cause perinatal cerebral hemorrhage [121]. Degradation of the ECM is central to BBB disruption in adult stroke [122]. Activation of MMPs, MMP-9, MMP-3, and MMP-2 in particular, plays important roles in mediating ECM breakdown, degradation of TJs, laminin, collagen, and fibronectin, leading to vasogenic edema, BBB leakage, and immune cell infiltration [123–128]. Infiltrating immune cells, microglia and activated astrocytes are major sources of MMPs after stroke [129, 130]. MMPs produced by activated astrocytes and neurons were also reported to promote repair [131, 132]. In rodent neonatal post-ischemic brain, upregulation of MMP-9 was observed as early as 24 h after injury ([133] and Fig. 3A). MMPs could also enable signaling of adhesion molecules and EC activation and be critical in monocyte trafficking and recruitment, forming post-ischemic inflammatory cascades in injured neonatal brain [134–136].

Pericyte number is low in neonatal brain, and their recruitment from vessels to newly formed capillaries is tightly synchronized during development [19]. Pericytes modulate blood flow resistance during development [137] and contribute to the BBB structure by producing many components of the basement membrane that surround ECs [19, 138, 139] and support angiogenesis, microvasculature stabilization, capillary diameter regulation, and clearance of toxic compounds [140]. In models of adult stroke, pericyte loss or dissociation from vessels causes edema, impairs CBF, and upregulates MMP-9 [141–146], leading to leukocyte extravasation and BBB breakdown, but there is essentially no information on the role of pericytes in the post-ischemic developing brain.

Astrocytes can maintain BBB function via direct physical interaction with brain cells through their end-feet, regulation of calcium signaling and CBF [147], as well as sustaining of signaling molecules, such as VEGF, GDNF, angiopoierin-1, and TGF-β [148, 149]. Upon injury, astrocyte activation is associated with gliosis and scar formation, production of cytokines, and other inflammatory events, such as MMPs activation, modulating the neurovasculature both by promoting and disrupting BBB functionality [131, 150–152]. Increased IL-15 production by astrocytes after neonatal HI has been linked to T and NK cell recruitment and aggravation of brain damage [153]. In co-cultures, astrocytes were also shown to have a direct cross-talk with microglia and to induce an anti-inflammatory phenotype [154]. Although astrocytes are rather resistant to ischemic injury, their increase of acquaporin-4 (Aqp-4) expression in the adult brain is associated with swelling due to impaired water fluxes and consequent brain edema [155]. These phenomena were, however, not observed in a neonatal stroke model, since vasogenic edema that usually characterizes injured regions was not apparent in brain areas with high expression of Aqp-4 [71]. Early after tMCAO in P7 rats, we observed disassociation of astrocytes from the vasculature and reduced Aqp-4 vascular coverage (Fig. 3B). The multifunctional capabilities of astrocytes during neuroinflammation make them intriguing candidates for maturation-specific therapeutic interventions during stroke in different developmental stages as reviewed elsewhere [156, 157].

## Neuroinflammation

**Fig. 4:**
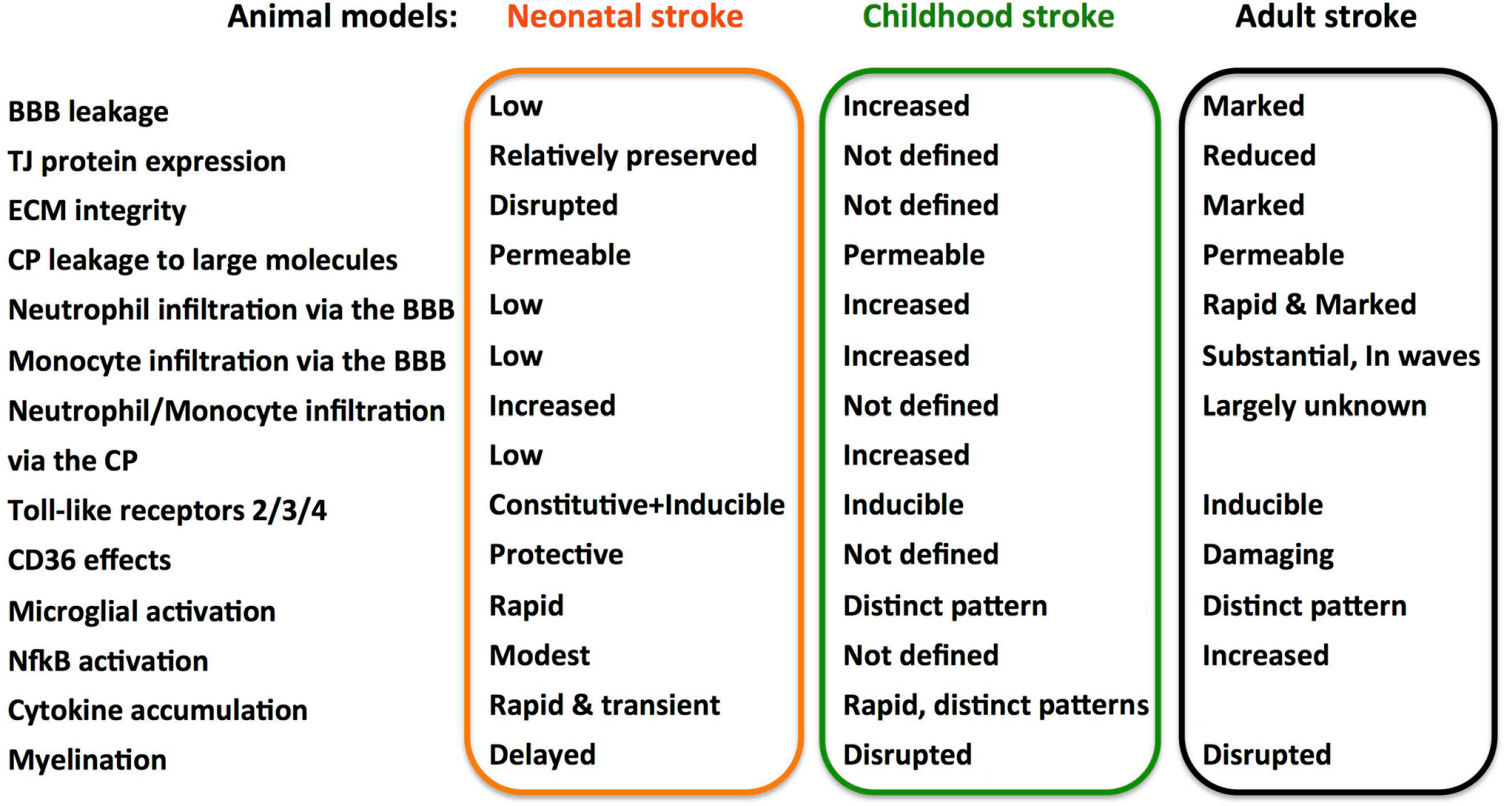
Brain maturation–dependent differences in individual mechanisms of acute injury in rodent models of PAIS, CAIS, and AIS

Neuroinflammation itself is a major contributor to neonatal and juvenile brain injury. It is mediated by several different cell types, both local and peripheral, which upon activation can release a plethora of signaling molecules [91, 158]. The contribution of inflammatory cascades and immune mediators, however, varies upon the developmental stage at the time of the insult (summarized in Fig. 4).

### Brain Immune Cells

Microglia are the primary immune cells in the brain under normal physiological conditions and represent 12–15% of the CNS cellular component. Microglia provide support for CNS development, including during phases of neurogenesis, angiogenesis, myelination, synaptogenesis, as well as govern synaptic pruning [159–161]. Upon injury, activated microglia have been traditionally considered toxic by releasing inflammatory mediators and ROS [162, 163], yet recent data in tMCAO models have demonstrated their protective role. In an adult tMCAO model, microglial depletion reduced the threshold of spreading depolarization and the related potassium uptake in the mouse brain [164], demonstrating that microglia preserve neuronal function through specialized somatic purinergic junctions [165]. Microglial depletion using intracerebral administration of liposome-encapsulated clodronate prior to tMCAO in neonatal rats and mice actually enhanced neuroinflammation, exacerbated brain injury, and triggered brain hemorrhages 24 h after stroke [166, 167] whereas BBB integrity is preserved in injured pups with unperturbed microglia (Fig. 3C). TGFβ signaling in microglial cells was shown to protect neonatal rat and mouse brains from hemorrhagic transformation after tMCAO [166]. This protective role is further supported by a recent study where microglia depletion before induction of neonatal HI aggravated brain injury and reduced expression of TGFβ and IL-10 [168]. Furthermore, microglia can modulate injury in concert with leukocytes, revealing the role of a specific microglia-leucocyte axis in PAIS [94, 169, 170].

Border-associated macrophages (BAMs) are another type of resident macrophages that populate the CP, meninges, and perivascular spaces and express both genes found in resident microglia and bone marrow-derived cells. About 30% of BAMs share genetic origins with monocytes that are suggested to repopulate the perivascular spaces after ischemia [171]. Specific transcriptome differences in BAMs at the meninges were identified in the developing brain, shedding new light on BAMs role under physiological or pathological conditions [113], but there are no data yet on the role of BAMs after ischemic stroke during different brain maturation stages.

Oligodendrocyte development and its implications in perinatal white matter injury and HIE have been recently reviewed elsewhere [172, 173].

### Peripheral Immune Cells

Neutrophils are well described as first responders to stroke [174–178]. Although neutrophil infiltration into the neonatal brain 1–24 h after tMCAO is negligible, they are chemoattracted through CINC-1/KC signaling [179] and accumulate at the level of CP, where they release cytokines and ROS, shortly after reperfusion [105, 108]. Comparisons of neutrophil accumulation within 24 h after tMCAO in P7–P9 [108] and P21 rodents [78] depicted more profound neutrophil accumulation into injured juvenile brain compared to neonatal brain and increased BBB albumin leakage. Importantly, neutrophil activation and recruitment to the brain are reported to be regulated via cross-talk with both microglia and monocytes via CCR2 signaling in monocytes. Juvenile mice deficient in CCR2 and CX3CR1 displayed lower neutrophil numbers, more preserved brain vasculature, and smaller infarct compared to wild type mice due to decreased accumulation of monocytes and macrophages within the ischemic brain [105, 180]. These results suggest that mechanisms that maintain BBB integrity after stroke during neonatal period undergo changes during postnatal development and that children may be more susceptible to peripheral immune-mediated brain damage following stroke compared to neonates.

Release of neutrophil extracellular traps (NETosis) is one of mechanisms of neutrophil-mediated injury in adult stroke, in part via HMGB1-mediated mechanisms [129, 181–184]. While this process is currently under investigation in the post-ischemic neonatal brain, administration of viral mimetic toll-like receptor (TLR) 3 agonist Poly-IC to juvenile mice rapidly activated neutrophil elastase and NET formation, inducing vascular leakage and immune cell recruitment to the cerebral vasculature. Importantly, pharmacological inhibition of neutrophil elastase prevented vascular distortions, revealing a potential novel therapy for arteriopathy-induced strokes in children [85]. Overall, these findings highlight potential key role of neutrophils in influencing CAIS.

Monocytes, that can be categorized as classical (Ly6^hi^CCR2^+^CX3CR1^mid^) and non-classical (Ly6^low^CCR2^−^CX3CR1^hi^) subtypes based on their phenotype and function, are recruited to the injury site where they differentiate into macrophages, phagocyte cellular debris, and take part in the inflammatory cascades [185, 186]. In neonates, monocytes have lower adhesion and antigen presentation capabilities compared to adults and thus are less capable of infiltrating into the developing brain [187] but have a greater capability for cytokine secretion, affecting inflammatory response [188]. Infiltration into the post-ischemic brain is thus age- and model-dependent and can play both beneficial and detrimental roles, depending on timing and the severity of injury [78, 189–191]. After neonatal tMCAO, monocytes are rapidly recruited via the CX3CR1-CCR2 axis and signal at the level of the CPs with limited transmigration into the brain parenchyma [105]. While only few CCR2 + monocytes are seen in the injured parenchyma within 24 h after tMCAO in neonatal mice, their numbers increase, while injury and endothelial cell death evolve. In models of neonatal HI brain injury, instead, monocytes and MDMs were shown to reach the injury site biphasically via a disrupted BBB, contribute to brain damage [191], and to become pathological microglia-like cells by maintaining a long-term inflammatory phenotype [192]. Transmigrated monocytes can gradually differentiate into microglia-like cells with ramified cell bodies in support of the local pool of resident cells [193].

In a mouse CAIS model, we demonstrated the presence of CCR2 + monocytes after acute injury and attenuated brain injury in mice deficient in CX3CR1/CCR2 signaling [78]. Along same lines, in human CAIS, differentiation of monocytes into a pro-inflammatory phenotype rather than a restorative phenotype impaired endothelial repair response genes, potentially making these children more susceptible to stroke recurrence [194]. Together, these data suggest a bidirectional role in monocyte-endothelial signaling in childhood stroke.

T cells mediate microvascular dysfunction in post-ischemic adult brain by producing MMPs, ROS, and pro-inflammatory factors that degrade ECM and damage ECs [195, 196]. In neonates, lymphocytes infiltrate post-ischemic brain for months after injury, suggesting their long-term contribution to the inflammatory response [197, 198]. Treatment with fingolimod (FTY720), an immunomodulatory drug clinically used for multiple sclerosis patients, was shown to reduce the number of circulating CD4^+^ and CD8^+^ cells in neonatal HI model, although data on the neuroprotective effects are conflicting [199, 200]. While pharmacological depletion of T cells after neonatal HI exacerbated brain injury and increased infiltration of innate immune cells into the brain parenchyma, deficiency of both B and T cells using Rag-/- mice reduced lesion size in a neonatal model of white matter injury [199, 201]. Interestingly, infiltration of regulatory T cells (T regs) into injured brain provided endogenous neuroprotection in female mice after neonatal HI, while T-reg interaction with brain vessels in male neonates after HI induce secondary neurodegerenation due to vascular injury [202]. As of now, the role of lymphocytes in PAIS and CAIS is still undefined.

Other immune cells such as mast cells and natural killer (NK) cells also affect stroke, but the number of studies on the role of these cells in neonatal brain injuries is limited. Activated mast cells were reported to be among the first responders after neonatal HI and focal stroke by undergoing degranulation, histamine release, and release TNF, contributing to neuronal death [203–205]. A role for spleen-associated NK cells in contributing to neonatal brain injury was demonstrated in neonatal HI model [153].

### Signaling Molecules and Receptors in Inflammation

Communication between different cell types is regulated by a variety of signaling molecules. Cytokines, chemokines, adhesion molecules, and MMPs are among key components involved in the inflammatory process [206, 207].

### Cytokines and Chemokines

The cellular source, types, and magnitude of produced cytokines and chemokines depend on injury severity, the type of insult, and timing after insult. For example, in neonatal HI, there was an increase of pro-inflammatory cytokines such as IL-1β, IL-6, and TNF-α in the blood and the CSF soon after the insult [208, 209], while presence of anti-inflammatory molecules like IL-10 was detected in the serum and brain of neonatal rats within hours after injury [210]. Expression of IL-6 and TNFα in astrocytes, microglia, or neurons attracts neutrophils, while release of IL-23 from microglia can contribute to recruitment of T cells [177]. Following tMCAO in P7 rats, there is marked robust (within 1–3 h after reperfusion) transient increase of IL-1β, IL-6, and MCP-1 in the plasma and a more delayed IL-1β, IL-6, and MCP-1 accumulation in ischemic-reperfused tissue [179]. Multiple cell types upregulated cytokine and chemokine production in injured regions [167], but there were no significant increase in TNFα levels in plasma or in injured region at 24 h. Similarly, in tMCAO model in P9–P10 mice, accumulation of only a subpopulation of cytokines and chemokines was observed, that did not include TNFα [65, 211]. Several immunomodulatory strategies have been shown to reduce inflammation and injury in HI model combined with LPS pre-treatment, such as administration of IL-1 receptor antagonist (IL-1RA) [212]. A number of pre- and post-conditional strategies have been tested, as summarized elsewhere [213].

In a CAIS model, accumulation of IL-6, TNFα, MCP-1, and KC was marked at 24 h, but there was no increase in IL-1β levels and IL-10 and IL-4 in injured regions [78]. CX3CR1 and CCR2 deficiency attenuated accumulation of chemokines MCP-1 and KC, changes that paralleled attenuated monocyte and neutrophil infiltration [78]. These data indicate age-dependent patterns of the inflammatory response to stroke.

Clinically, children with abnormal neurodevelopmental outcome following neonatal encephalopathy have higher peripheral levels of several cytokines and chemokines [33]. Similarly, increased cytokine and chemokine levels are reported in the CSF in children with cerebral arteriopathies [214] and neonates following birth asphyxia [209].

### Receptors

Pattern recognition receptors (PRRs), including TLR receptors, play a key role across ages in many diseases, including stroke and infection (reviewed in [91, 215, 216]). PRRs are expressed in multiple immune cells and mediate synthesis of inflammatory molecules like IL-1β, TNF-α, iNOS, and COX2 [217], contributing to progression of cell death. Activation of TLR1/2/3 increases the vulnerability of the neonatal brain to HI [217, 218], in part by mediating leukocyte trafficking to the developing brain through the CP [219]. There are major differences in TLR2 expression after tMCAO between adult and neonatal brain, a rapid marked increase in expression following adult tMCAO [220] and only marginal difference following neonatal tMCAO [221]. In neonates, increased immune cell trafficking via the CP ipsilateral to the MCAO occurs in a TLR2-dependent manner [105] and increases BCSFB permeability, thus suggesting that post-ischemic inflammatory patterns in the neonatal brain are stimuli-dependent upon TLR activation and, more importantly, through communication with other receptors [219]. Our studies also revealed more than 30 fold decrease in TLR2 expression between P8 and P28 under physiological conditions [221], making age-dependent TLR2 contribution to stroke likely.

CC and CXC receptors, receptors that attract monocytes and neutrophils, respectively, are upregulated in PAIS and CAIS models [78, 108, 167]. As we already discussed, KC/CINC-1-dependent neutrophil infiltration is low in PAIS model [108] but is increased in a CAIS model [78]. The injurious role of CCR2 and CX3CR1 became evident by attenuated acute injury in mice with disrupted receptors. Of note, neutrophil infiltration was attenuated as well, demonstrating an interplay between monocyte and neutrophil signaling after neonatal stroke.

The scavenger receptor CD36 is central to multiple biological functions in ECs, microglia, and monocytes, including uptake of long-chain fatty acids and oxLDL, phagocytosis of apoptotic debris, and cell chemotaxis [222–225]. Via multiple ligands (phospholipids, advanced glycation end products, etc.) and partnering with multiple receptors in the lipid fraction (including TLR2/4/6, LOX) [226], CD36 serves as “master switch” in assembling and triggering inflammatory pathways and ROS production. CD36 is injurious after acute adult stroke [65, 227, 228] but protective in acute perinatal stroke by phagocytosis apoptotic debris [229]. Such a rather opposing maturation-dependent response in part depends on availability of ligands in neonatal vs. adult brains as well as the recruitment of both neutrophils and monocytes at the level of the CP, influencing the metabolic and ECM signaling [230]. CD36 function thus differs depending on brain maturation, and further studies are needed to define age-dependent effects in the developing brain.

An array of other receptors play fundamental roles after stroke. For example, microglial expression of purinergic receptors P2X and P2Y (P2RY12) was shown to promote microglia-vessel interactions to maintain BBB closure after cerebrovascular damage [231] and to protect neuronal functions [165]. Also, TGFBR2 in endothelial cells was shown to modulate vascular sprouting during fetal development [232] and TGFBR2 signaling in microglial cells to protect BBB integrity in PAIS [166]. The role of dynamic neurodevelopment changes in expression of these receptors in relation to their impact on stroke pathology in different age groups is yet to be understood.

## Sex Differences

Neonatal and childhood stroke are sexually dimorphic, in large due to a complex interplay between sex- and sex hormone-related immune activation in the brain, as evident in humans and in animal models [33, 100, 233–235]. Male neonates are reported to be more susceptible than female neonates and have worse outcomes compared to females with similar injury [236]. Although the nature of the specific mechanisms explaining such differences between males and females is far from being understood, several biological processes have been identified, including higher susceptibility to oxidative stress and enhanced microglial responses in the male brain [100, 237]. Microglia are reported to be sexually dimorphic in the neonatal brain under physiological conditions, with differences evident in microglial cell morphology, transcriptional state, and functionality [237–240]. Recent findings have also highlighted opposite functions of Treg after neonatal HI [202], suggesting different neuroinflammatory responses after injury. Sex-dependent effects of inflammation in neonatal brains were also identified to have implications for neuro-psychiatric disorders [241]. Thus, consideration to sex dependence should be given while developing therapeutic approaches for infants and children.

## Brain Repair After Stroke in Immature Rodents

Research over the past decade pointed out the need to focus on long-term brain repair, especially given only short-term success of various therapeutic approaches in neonatal HI and arterial stroke models [91]. While literature is extensive of neural repair in neonatal HI, long-term studies in PAIS model are sparse [242]. One important observation made in neonatal rat tMCAO is based on the assumption that angiogenesis and repair should take off early after injury and be more profound in neonatal than in adult stroke due to developmental plasticity. Nonetheless, while ECs proliferation occurs in P7 [88] and P10 rats [243] subjected to tMCAO, the number of proliferating ECs was significantly reduced in the core and perifocal lesions up to 2 weeks after tMCAO in neonatal rats [88]. Conversely, the angiogenic response following adult stroke was reported within 24 h after tMCAO [244–246]. While there were no follow-up studies to fully understand this unexpected injury pattern, brain plasticity can be controlled at the molecular and network level at different developmental stages and depend on the state of the neurovascular unit, perhaps its low permissiveness, adversely affecting the recovery of functional neuronal circuits [247]. Growth factors (i.e., VEGF and BDNF) that are needed in the peri-infarct areas for cell migration towards the penumbra [248, 249] and promoting both angiogenesis and proliferation in neurogenic areas, such as SVZ and hippocampus, are present in both neonatal and adult stroke [250–253], thus, making it unlikely to be limiting repair. However, the endogenous repair mechanisms in the post-ischemic brain can also be limited as the differentiation of proliferative cells into astrocytes rather than neurons and OLs can contribute to astrogliosis to inhibit neural repair and brain connectivity [254]. As discussed below, cell therapies and growth factor administration could enhance the repair.

## Therapies

Collateral blood flow is rather extensive in the developing brain [86] and the penumbra, which has long been the pharmacological target for acute ischemic stroke treatment [255], observed on MRI in more than half neonates after PAIS, giving hope for development effective therapies. Although several strategies are being considered for cerebral palsy and stroke (reviewed in [256]), there are essentially no effective therapies that protect the developing brain through adulthood, and therapeutic approaches meant at attenuating effects of cerebral ischemia in neonates have proven to be short-lived, demonstrating the need to focus on neural repair after neonatal stroke.

Therapeutic hypothermia (HT) is the only approved treatment for HIE that offers neuroprotection and reduces long-term disability [257]. The beneficial role of HT, however, is limited to milder HIE cases, and its applicability to neonatal arterial stroke has not been clearly demonstrated. The premise of HT is to delay cell membrane depolarization and attenuate intracellular Ca2 + influx and extracellular glutamate release [258]. HT also modulates the inflammatory pathways, including reducing pro-inflammatory cytokine production and attenuating microglia proliferation [259–261]. At the same time, studies in humans and in animal models have revealed the need to better understand re-warming patterns to limit unwanted adverse effects. Several pharmacological strategies geared towards promoting neurogenesis have also been tested in clinical trials after promising preclinical outcomes, but with little success as of now. For example, erythropoietin treatment in neonatal rats after tMCAO induced neurogenesis and oligodendrogenesis and offered long-term neuroprotection [262, 263], even when treatment was delayed to 72 h after injury [264], but clinical efficacy was not demonstrated [265], at least in preterm babies (reviewed in [266]). Recent studies have also suggested PJ34, the poly(ADP-ribose) polymerase inhibitor, and SAG, the Shh-Smoothened agonist, to be neuroprotective after MCAO in neonatal rodents [267, 268].

During the past decades, cell-based therapies have provided encouraging results preventing perinatal brain injuries or enhancing repair in experimental settings*,* as recently reviewed in detail elsewhere [253, 269–271]. Administration of umbilical cord blood cells (UCBCs), for instance, inhibited microglial activation following neonatal HI [272], attenuated reactive gliosis and reduced infiltration of leukocytes into the brain, and supported BBB function [273]. Mesenchymal stem cells (MSCs) were shown to promote regeneration and reduce gliosis when administered intranasally 10 days after HI [274]. Similarly, MSC administration in P10 rats after tMCAO improved long-term functional outcomes and provided white matter protection when administered 3 days after injury [74, 275]. Of interest, the first human study has recently shown the feasibility of intranasal administration of MSCs without serious adverse effects, suggesting the safety of the treatment and route of administration [276]. Studies in neonatal HI model showed that transplanted cells are short-lived and become undetectable soon after administration [277], but they affect local environment, oligodendrogenesis, and myelination. MSCs were shown to promote angiogenesis, increase neurovascular remodeling, and improve neurogenesis and neurological outcomes by rewiring neuronal circuitry after stroke by releasing extracellular vesicles (EVs), exosomes in particular [278, 279]. In tMCAO in P9 mice MSCs-derived EVs were shown to act via modulatory effects on microglial cells and ECs [280]. Transfer of microvesicles, but not exosomes, improved mitochondrial function in EC cultures subjected to oxygen–glucose deprivation. EC-derived EVs resulted in a greater extent of energy transfer compared to macrophage-derived EVs, demonstrating EVs potential to maintain healthy ECs and prevent alteration of BBB functionality after stroke [281]. Cell modulation via EVs could thus represent a novel potentially powerful therapeutic alternative to cell-based treatments, but efficacy and safety are yet to be demonstrated in clinical trials.

In children, given the influence of infection-induced immune activation that contributes to arteriopathies and vascular dysfunction-induced stroke, steroid treatments have been utilized. While administration of steroids could be beneficial for limiting infection-induced arteriopathies, more data is required to show that they can either treat or prevent arteriopathy-induced stroke by muting the immune response [48]. Given the high association between infections and stroke in children, another line of investigation is focused on quelling infection-induced immunity [282]. Based on these data, one possible preventive approach is vaccine administration that would promote immune quiescence and induce resilience of cerebral arteries to infection and associated strokes. Finally, preconditioning, a phenomenon that consists of induction of sublethal stress (i.e., hypoxia, ischemia) or drug administration before the main ischemic event, as well as post-conditioning, meant to induce resistance to a subsequent potentially lethal ischemic insult, has been demonstrated to be safe and is being actively studied in adult animal stroke models and in small stroke clinical trials [283–285]. This therapeutic approach may also prove beneficial in children.

Overall, therapies for both reducing the outcomes and/or preventing the incidence of neonatal and childhood stroke are lacking. Thus, it is crucial that more preclinical studies that reveal age-specific stroke mechanisms and more clinical studies that identify risk factors and biomarkers for ischemic brain disease are conducted.

## Summary and Conclusions

It is no longer disputed that the developmental stage of the brain at stroke onset plays a key role in injury and that inflammation is an established hallmark of brain injury in infants and children. Establishment of PAIS and CAIS models enabled identification of several brain-maturation mechanisms of injury, findings that we discuss in this review. However, substantial gaps exist in the understanding of the pathophysiology of PAIS and CAIS. An improved understanding of the specific cellular and molecular pathways involved in the post-ischemic cascades during different stages of brain maturation will help identify brain maturation-specific targets and therapies for newborns and children who suffer stroke.

## Data Availability

Data from the Vexler laboratory relevant to the topics discussed in this review is available.
